# Comparison of Endarterectomy and Stenting in the Treatment of Carotid Artery Stenosis: A Real-World Nationwide, Total Population-Based Study from Korea

**DOI:** 10.5761/atcs.oa.24-00177

**Published:** 2025-05-01

**Authors:** Sang Ah Lee, Dong-Hyuk Cho, Jimi Choi, Jun Gyo Gwon

**Affiliations:** 1 Department of Surgery, Division of Vascular Surgery, University of Ulsan College of Medicine and Asan Medical Center, Seoul, Korea; 2 Division of Cardiology, Department of Internal Medicine, Korea University Anam Hospital, Korea University College of Medicine, Seoul, Korea; 3 Department of Internal Medicine, Division of Endocrinology and Metabolism, Anam Hospital, Korea University College of Medicine, Seoul, Korea

**Keywords:** endarterectomy, carotid, endovascular procedures, stroke, carotid stenosis

## Abstract

**Purpose:** Carotid endarterectomy (CEA) and carotid artery stenting (CAS) are both well-established treatments for carotid artery stenosis. We analyzed real-world data from the Korean National Health Insurance Service (NHIS) database to compare the clinical outcomes.

**Methods:** This retrospective cohort study included patients with carotid artery stenosis registered in the NHIS from 2008 to 2018. Patients who underwent either treatment were divided into CEA or CAS groups and subjected to 1:4 propensity score matching.

**Results:** The study cohort included 1521 CEA and 6768 CAS patients. In symptomatic patients, the stroke rate within 1 month was lower in the CAS group (hazard ratio [HR], 0.61). However, the incidence of cardiovascular disease (CVD) death was higher in the CAS group at 1 month, 1 year, and during the total follow-up (HRs, 4.18, 2.43, and 1.50). There were no significant differences in outcomes between asymptomatic patients in the 2 groups.

**Conclusion:** The periprocedural stroke risk was higher in symptomatic carotid stenosis patients who underwent CEA, but mortality was higher in those who received CAS, both in the short and long term. In asymptomatic patients, however, the incidence of major adverse cardiovascular events and mortality was similar between the 2 groups.

## Introduction

There are 7.6 million ischemic strokes reported globally each year, resulting in 3.3 million deaths.^1)^ A prior study from the United States involving 2204 ischemic stroke patients reported that large artery atherosclerosis was the underlying cause in 16.6% of cases and that an ipsilateral 50%–99% carotid stenosis could be identified in 8% of this population.^2)^ Carotid endarterectomy (CEA) and carotid artery stenting (CAS) are both established as effective treatments for symptomatic and asymptomatic carotid artery stenosis.^3,4)^ Multiple studies have compared the outcomes of these 2 approaches. A previous systematic review of 22 randomized controlled trials (RCTs) showed that stenting for symptomatic carotid stenosis is associated with a higher risk of periprocedural stroke or death compared to endarterectomy.^5)^ However, few other reports to date have compared the clinical results of carotid revascularization for carotid stenosis in an Asian population.

The number of carotid revascularization procedures used to treat carotid stenosis is relatively lower in Korea than in Western countries, including the United States and Europe. A prior study calculated that 2800 carotid revascularization procedures were conducted in Korea in 2013, comprising 587 CEA and 2312 CAS cases.^6)^ A study from the United Kingdom, which has a population 1.3 times greater than Korea, described 4356 cases of CEA in that same year.^7)^ When comparing the rate of carotid revascularization procedures per population, the total carotid revascularization rate in Korea was 57 procedures per 1000000 persons in 2013, with CEA accounting for 11 per 1000000 persons. In contrast, based on population estimates, the CEA rate in the United Kingdom in 2013 was approximately 86 per 1000000 adults (aged 16 and older).^8)^ This stark difference highlights the lower utilization of overall carotid revascularization procedures, especially CEA, in Korea.

It is well established that the incidence of cardiovascular disease (CVD) is lower in East Asian countries than among Western populations.^9)^ We speculated, therefore, that the clinical outcomes of carotid revascularization procedures might be different between these populations. Our objective was to examine real-world data on carotid revascularizations and evaluate the clinical outcomes of CEA and CAS in a Korean cohort from the Korean National Health Insurance Service (NHIS) database.

## Materials and Methods

### Study design and database

**Fig. 1:**
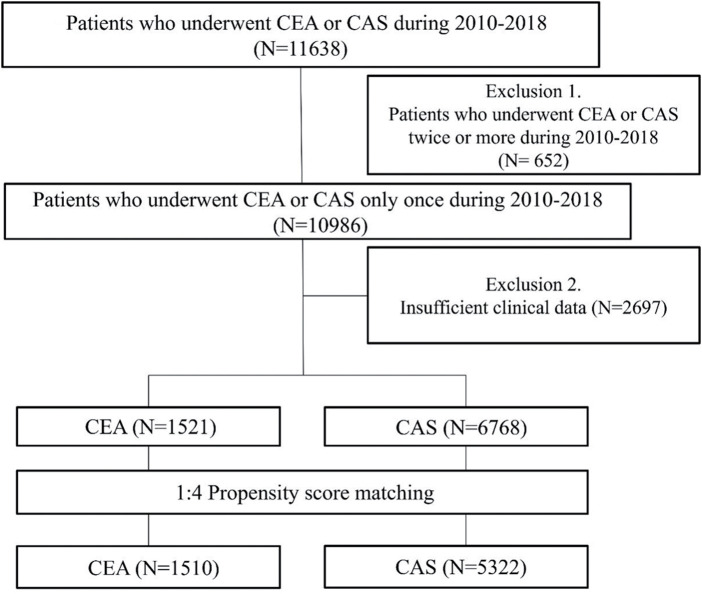
Study flowchart for the comparison of patients who underwent CEA or CAS. CEA: carotid endarterectomy; CAS: carotid artery stenting

This was a retrospective cohort study involving patients registered with the NHIS between 2008 and 2018. Patients who underwent CEA or CAS procedures during this period were included. Subjects who underwent CEA or CAS during the washout period (2008–2009) were excluded. Patients who received CEA or CAS 2 or more times during the study period, as well as those without clinical data in the NHIS database, were also excluded from the analysis (**Fig. 1**). Patient age, sex, alcohol consumption, cholesterol levels, medications, and diseases according to the 10th edition of the International Classification of Diseases Diagnostic Code (ICD-10) were investigated. This study was conducted in accordance with the principles of the Declaration of Helsinki, with approval from the Ethics Committee of Korea University Anam Hospital (2020AN0279).

### Study subjects and clinical outcomes

The included study patients were divided into CEA and CAS groups and subjected to 1:4 propensity score matching. The clinical outcomes of the 2 groups were then compared. CEA patients were defined as those entered under NHIS CEA codes O0226, O0227, O2066, and O2063. For CAS cases, the identifying code was M6602. Patients with ischemic stroke, stroke, and transient ischemic attack were defined as those assigned ICD-10 codes I63, I60–I64, and G45, who underwent a computed tomography scan or magnetic resonance imaging at the time they were assigned a disease code. Patients with myocardial infarction (MI) were defined by ICD-10 codes I21–I22, indicating that they underwent hospitalization and coronary artery angiography. CVD death was defined by ICD-10 codes ranging from I00 to I99 as the cause of death. Major adverse cardiovascular events (MACE) were defined as coronary artery disease, stroke, or CVD death. Symptomatic patients were defined as those hospitalized with ischemic stroke or transient ischemic attack within 180 days prior to carotid revascularization. The use of specific drugs was defined as medication prescribed within 1 month of the procedure.

### Statistical analysis

Data are presented as means ± standard deviations or as median values (interquartile range) for continuous variables, and as numbers (percentages) for categorical variables. Demographic and clinical characteristics were compared by calculating the standardized mean difference (SMD) between the study groups. We derived propensity scores from a multiple logistic regression model that included sex, age, smoking status (current, former, or never), alcohol consumption (≥1 or <1 times/week), body mass index (BMI), high-density lipoprotein cholesterol, low-density lipoprotein cholesterol, and triglycerides, as well as comorbidities such as diabetes mellitus, hypertension, atrial fibrillation, dyslipidemia, peripheral artery disease, or ischemic heart disease, symptomatic or asymptomatic status, and the use of statins, anticoagulants, or antiplatelet agents. All SMDs between the 2 groups after propensity matching were 0.1 for baseline covariates, except for the symptomatic or asymptomatic status. The cumulative incidence of outcomes was graphically presented using a Kaplan–Meier curve. A Cox proportional hazards regression model with a robust sandwich variance estimator was used to evaluate the relationship between group and study outcomes, accounting for correlations within matched clusters. Symptomatic or asymptomatic status was included as an adjusting factor in the model. The risk of outcomes in the CAS group compared with the CEA group was presented as a hazard ratio (HR) and 95% confidence interval (CI). The same model was used to assess outcomes according to symptomatic or asymptomatic status. Baseline characteristics for subgroup analysis included sex, prior ischemic heart disease, use of anticoagulants, and age (≥75 and <70 years).

All statistical analyses were performed using SAS Enterprise Guide software, version 7.1 (SAS Institute Inc., Cary, NC, USA), and a 2-sided P value of <0.05 was considered statistically significant.

## Results

### Study population and characteristics

A total of 10986 patients in Korea were registered as having undergone CEA or CAS once during the study period. After excluding cases with insufficient clinical data, 1521 patients received CEA, and 6768 patients received CAS. The median follow-up period was 44 months (interquartile range, 25.1–70.5). The 2 groups differed in terms of smoking, dyslipidemia, statin medication, and the proportion of symptomatic patients in the procedure indication. In the CEA group, 28.8% of the patients were asymptomatic, whereas this proportion was only 18.9% in the CAS group (P <0.001, SMD 0.234). After propensity score matching, no statistically significant differences were found between the 2 groups in any variables, except for the proportion of symptomatic patients in the procedure indication (**Table 1**).

**Table 1:** Baseline characteristics of the study patients who underwent CEA or CAS

Mean ± SD or n (%)	Before PS matching			After PS matching		
	CEA (N = 1521)	CAS (N = 6768)	SMD	CEA (N = 1510)	CAS (N = 5322)	SMD
Male	1321 (86.9)	5744 (84.9)	0.057	1311 (86.8)	4606 (86.5)	
Age (years)	68.0 ± 86.9	68.5 ± 9.3	0.050	68.0 (8.4)	68.3 (9.2)	0.027
Smoking			0.110			0.043
Never	533 (32.5)	2500 (36.9)		529 (35.0)	1900 (35.7)	
Ex-smoker	495 (32.4)	1928 (28.5)		489 (32.4)	1623 (30.5)	
Current	493 (36.9)	2340 (34.6)		492 (32.6)	1799 (33.8)	
Alcohol consumption			0.019			0.011
<1 time per week	828 (54.4)	3748 (55.4)		822 (54.4)	2926 (55.0)	
≥1 time per week	693 (45.6)	3020 (44.6)		688 (45.6)	2396 (45.0)	
Body mass index (kg/m^2^)	23.9 ± 2.9	23.9 ± 2.9	0.010	23.9 ± 3.0	23.9 ± 2.9	0.009
HDL-cholesterol (mg/dL)	50.0 ± 15.6	49.7 ± 13.5	0.023	50.0 ± 15.6	49.8 ± 13.1	0.017
LDL-cholesterol (mg/dL)	111.2 ± 41.2	113.6 ± 41.8	0.058	111.2 ± 40.7	112.1 ± 41.4	0.022
Triglyceride (mg/dL), median (IQR)	127 (90–185)	132 (93–190)	0.073	127 (90–185)	130 (90–190)	0.015
Comorbidity						
Diabetic mellitus	561 (36.9)	2434 (36.0)	0.019	558 (37.0)	1958 (36.8)	0.003
Hypertension	1261 (82.9)	5493 (81.2)	0.046	1252 (82.9)	4376 (82.2)	0.018
Atrial fibrillation	139 (9.1)	486 (7.2)	0.072	132 (8.7)	408 (7.7)	0.039
Dyslipidemia	1366 (89.8)	5821 (86.0)	0.117	1356 (89.8)	4808 (90.3)	0.018
Peripheral artery disease	588 (38.7)	2632 (38.9)	0.005	584 (38.7)	2060 (38.7)	0.001
Ischemic stroke or transient ischemic attack			0.234			0.153
Asymptomatic	438 (28.8)	1279 (18.9)		431 (28.5)	1167 (21.9)	
Symptomatic	1083 (71.2)	5489 (81.1)		1079 (71.5)	4155 (78.1)	
Ischemic heart disease	647 (42.5)	2559 (37.8)	0.097	639 (42.3)	2170 (40.8)	0.031
Medication						
Statins	1422 (93.5)	6092 (90.0)	0.127	1412 (93.5)	5013 (94.2)	0.029
Anticoagulants	422 (27.7)	1922 (28.4)	0.015	417 (27.6)	1442 (27.1)	0.012
Antiplatelets	1516 (99.7)	6756 (99.8)	0.030	1506 (99.7)	5319 (99.9)	0.052

SD: standard deviation; CEA: carotid endarterectomy; CAS: carotid artery stenting; SMD: standardized mean difference; HDL: high-density lipoprotein; LDL: low-density lipoprotein; IQR: interquartile range

### Clinical outcomes

**Fig. 2:**
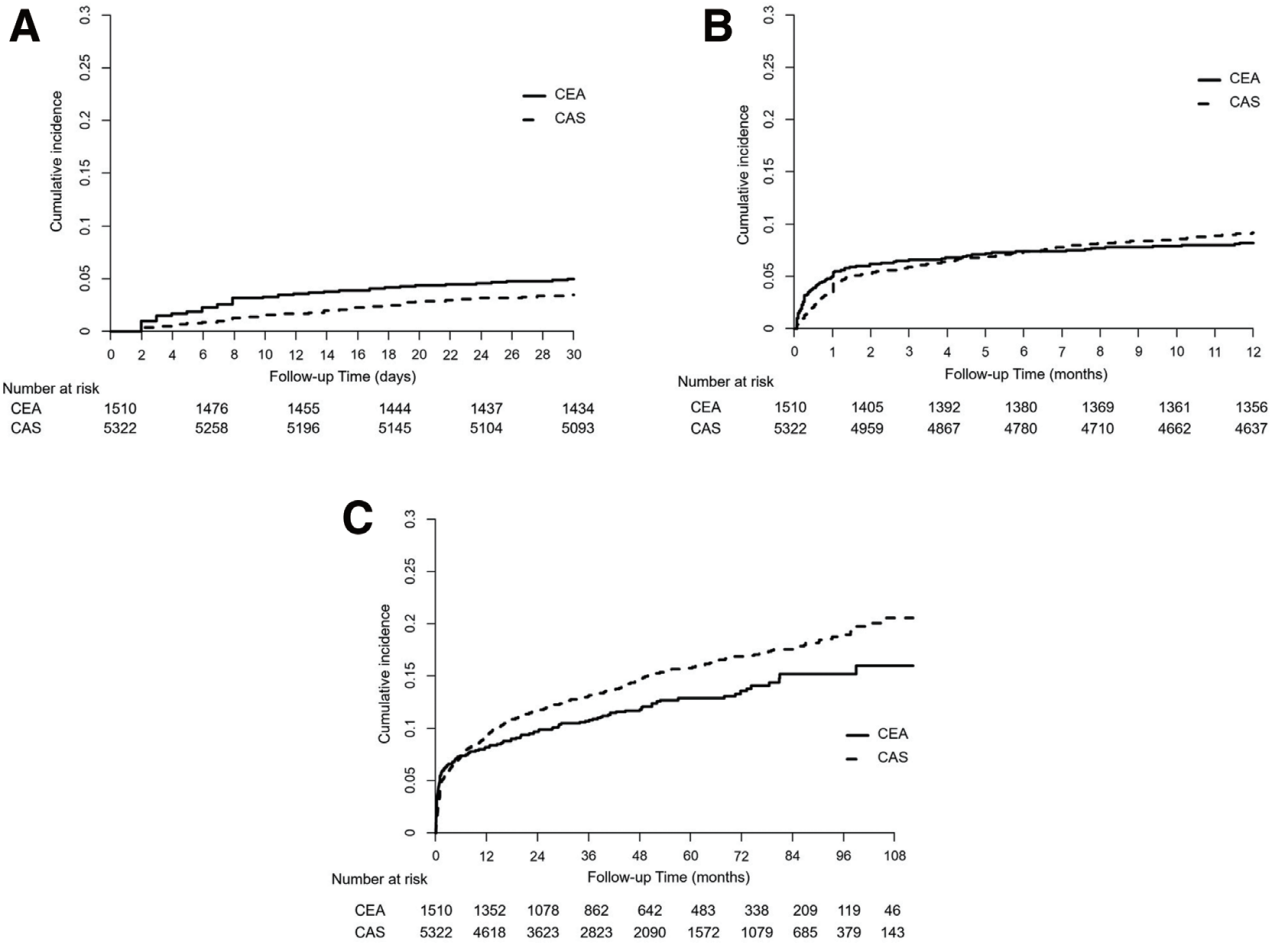
Stroke incidence in the total study cohort of patients who underwent carotid revascularization. (**A**) Cumulative incidence of stroke in CEA patients (solid line) and CAS patients (dotted line) within 1 month. (**B**) Cumulative incidence of stroke in CEA patients (solid line) and CAS patients (dotted line) within 1 year. (**C**) Cumulative incidence of stroke in CEA patients (solid line) and CAS patients (dotted line) during the total follow-up period. CEA: carotid endarterectomy; CAS: carotid artery stenting

The stroke incidence was lower in the CAS group within 1 month of the procedure (HR, 0.69; 95% CI, 0.53–0.89; P = 0.005). During the total follow-up period, however, the stroke incidence was higher among the CAS cases (HR, 1.23; 95% CI, 1.04–1.44; P = 0.016). The stroke incidence was 5.0% and 3.4% for the CEA and CAS groups within 1 month of the procedure and 11.9% and 14.3% in the total follow-up period, respectively (**Fig. 2**). The incidence of CVD death was higher among CAS patients both within 1 month of the procedure and during the total follow-up period (HR, 3.49; 95% CI, 1.26–9.68; P = 0.016 and HR, 1.49; 95% CI, 1.16–1.92; P = 0.002, respectively). All-cause mortality was also higher in the CAS group within 1 month of the procedure and during the total follow-up period (HR, 2.44; 95% CI, 1.16–5.35; P = 0.026 and HR, 1.18; 95% CI, 1.02–1.36; P = 0.025, respectively) (**Table 2**).

**Table 2:** Clinical outcomes including MACE and all-cause death after CAS or CEA

	CEA (n = 1510) (Reference)	CAS (n = 5322)	Hazard ratio (95% CI)	P
	Event (%)	Event (%)		
Within 1 month				
MACE	77 (5.1)	235 (4.4)	0.86 (0.67, 1.11)	0.239
MI	0 (0)	7 (0.1)	—	
Stroke	75 (5.0)	183 (3.4)	0.69 (0.53, 0.89)	0.005
CVD death	4 (0.3)	49 (0.9)	3.49 (1.26, 9.68)	0.016
All-cause death	7 (0.5)	60 (1.1)	2.44 (1.16, 5.35)	0.026
Within 1 year				
MACE	140 (9.3)	625 (11.7)	1.27 (1.06, 1.53)	0.011
MI	5 (0.3)	28 (0.5)	1.61 (0.62, 4.21)	0.328
Stroke	123 (8.1)	479 (9.0)	1.11 (0.91, 1.35)	0.318
CVD death	21 (1.4)	168 (3.2)	2.29 (1.45, 3.63)	<0.001
All-cause death	46 (3.0)	279 (5.2)	1.74 (1.28, 2.38)	0.001
Overall				
MACE	244 (16.2)	1065 (20.0)	1.27 (1.11, 1.47)	0.001
MI	28 (1.9)	81 (1.5)	0.86 (0.56, 1.32)	0.486
Stroke	180 (11.9)	760 (14.3)	1.23 (1.04, 1.44)	0.016
CVD death	71 (4.7)	363 (6.8)	1.49 (1.16, 1.92)	0.002
All-cause death	224 (14.8)	898 (16.9)	1.18 (1.02, 1.36)	0.025

CEA: carotid endarterectomy; CAS: carotid artery stenting; CI: confidence interval; MACE: major adverse cardiovascular event; MI: myocardial infarction; CVD: cardiovascular disease

### Symptomatic and asymptomatic patients

**Fig. 3:**
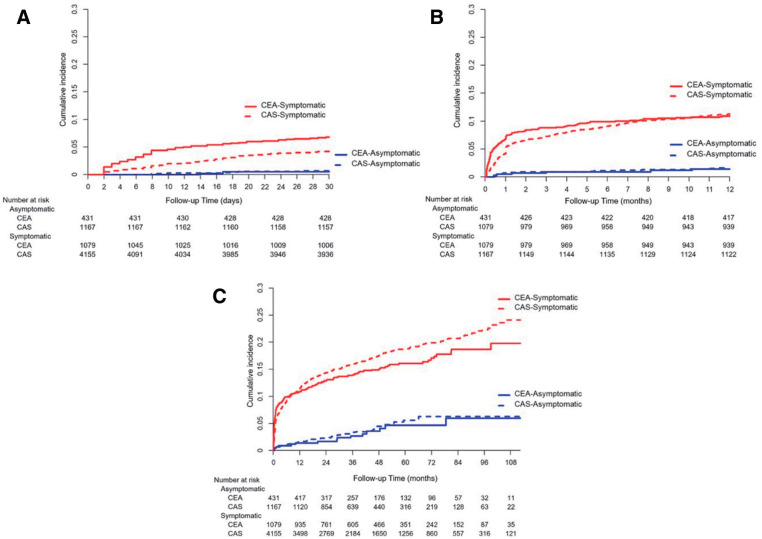
Stroke incidence in symptomatic and asymptomatic patients who underwent carotid revascularization. (**A**) Cumulative incidence of stroke in symptomatic (red solid line) and asymptomatic (blue solid line) CEA patients and symptomatic (red dotted line) and asymptomatic (blue dotted line) CAS patients within 1 month. (**B**) Cumulative incidence of stroke in symptomatic (red solid line) and asymptomatic (blue solid line) CEA patients and symptomatic (red dotted line) and asymptomatic (blue dotted line) CAS patients within 1 year. (**C**) Cumulative incidence of stroke in symptomatic (red solid line) and asymptomatic (blue solid line) CEA patients and symptomatic (red dotted line) and asymptomatic (blue dotted line) CAS patients within the total follow-up period. CEA: carotid endarterectomy; CAS: carotid artery stenting

Our study patients were further divided into symptomatic and asymptomatic groups. No differences were observed in the clinical outcomes between the symptomatic cases in the CEA and CAS groups, except for stroke occurrence within 1 month, which was lower in the CAS group (HR, 0.61; 95% CI, 0.47–0.80; P < 0.001). The stroke rates were 6.8% and 4.2% for CEA and CAS, respectively. There was no difference in stroke occurrence between CEA and CAS in the 1-year outcome and total follow-up periods (**Fig. 3**). The incidence of CVD death among symptomatic patients was higher in the CAS group than in the CEA group at all time points, including 1 month, 1 year, and over the entire follow-up period (HR, 4.18; 95% CI, 1.30–13.43; P = 0.016 at 1 month; HR, 2.43; 95% CI, 1.47–4.03; P = 0.001 at 1 year; and HR, 1.50; 95% CI, 1.14–1.97; P = 0.004 for the total follow-up; **Table 3**).

**Table 3:** Clinical outcomes including MACE and all-cause death after CAS or CEA stratified by the presence of symptoms

	Asymptomatic				Symptomatic				P for interaction
	CEA (n = 431)	CAS (n = 1167)	HR (95% CI)	P	CEA (n = 1079)	CAS (n = 4155)	HR (95% CI)	P	
	Event (%)	Event (%)			Event (%)	Event (%)			
Within 1 month									
MACE	2 (0.5)	13 (1.1)	2.40 (0.59, 9.78)	0.220	75 (7.0)	222 (5.3)	**0.76 (0.58, 0.98)**	**0.037**	0.113
MI	0 (0)	4 (0.3)	—		0 (0)	3 (0.1)	—		—
Stroke	2 (0.5)	8 (0.7)	1.47 (0.37, 5.87)	0.582	73 (6.8)	175 (4.2)	**0.61 (0.47, 0.80)**	**<.001**	0.223
CVD death	1 (0.2)	1 (0.1)	0.37 (0.02, 5.89)	0.481	3 (0.3)	48 (1.2)	**4.18 (1.30, 13.43)**	**0.016**	0.114
All-cause death	2 (0.5)	2 (0.2)	0.37 (0.05, 2.62)	0.319	5 (0.5)	58 (1.4)	**3.03 (1.21, 7.56)**	**0.017**	0.056
Within 1 year									
MACE	9 (2.1)	32 (2.7)	1.32 (0.64, 2.73)	0.459	131 (12.1)	593 (14.3)	1.17 (0.97, 1.42)	0.111	0.760
MI	1 (0.2)	5 (0.4)	1.85 (0.20, 17.01)	0.587	4 (0.4)	23 (0.6)	1.52 (0.53, 4.41)	0.439	0.877
Stroke	6 (1.4)	18 (1.5)	1.11 (0.46, 2.69)	0.818	117 (10.8)	461 (11.1)	1.02 (0.83, 1.25)	0.865	0.852
CVD death	4 (0.9)	11 (0.9)	1.02 (0.32, 3.20)	0.980	17 (1.6)	157 (3.8)	**2.43 (1.47, 4.03)**	**0.001**	0.172
All-cause death	10 (2.3)	31 (2.7)	1.14 (0.56, 2.32)	0.709	36 (3.3)	248 (6.0)	**1.82 (1.28, 2.58)**	**0.001**	0.251
Overall									
MACE	28 (6.5)	89 (7.6)	1.21 (0.79, 1.85)	0.374	216 (20.0)	976 (23.5)	**1.20 (1.03, 1.40)**	**0.019**	0.967
MI	5 (1.2)	21 (1.8)	1.67 (0.62, 4.51)	0.312	23 (2.1)	60 (1.4)	0.71 (0.44, 1.14)	0.155	0.126
Stroke	15 (3.5)	46 (3.9)	1.16 (0.65, 2.06)	0.613	165 (15.3)	714 (17.2)	1.14 (0.96, 1.36)	0.134	0.961
CVD death	12 (2.8)	102 (8.7)	1.03 (0.52, 2.01)	0.941	59 (5.5)	796 (19.2)	**1.50 (1.14, 1.97)**	**0.004**	0.305
All-cause death	46 (10.7)	32 (2.7)	0.87 (0.62, 1.22)	0.415	178 (16.5)	331 (8.1)	**1.20 (1.02, 1.41)**	**0.025**	0.089

CEA: carotid endarterectomy; CAS: carotid artery stenting; CI: confidence interval; MACE: major adverse cardiovascular event; MI: myocardial infarction; CVD: cardiovascular disease

### Variables affecting clinical outcomes

**Fig. 4:**
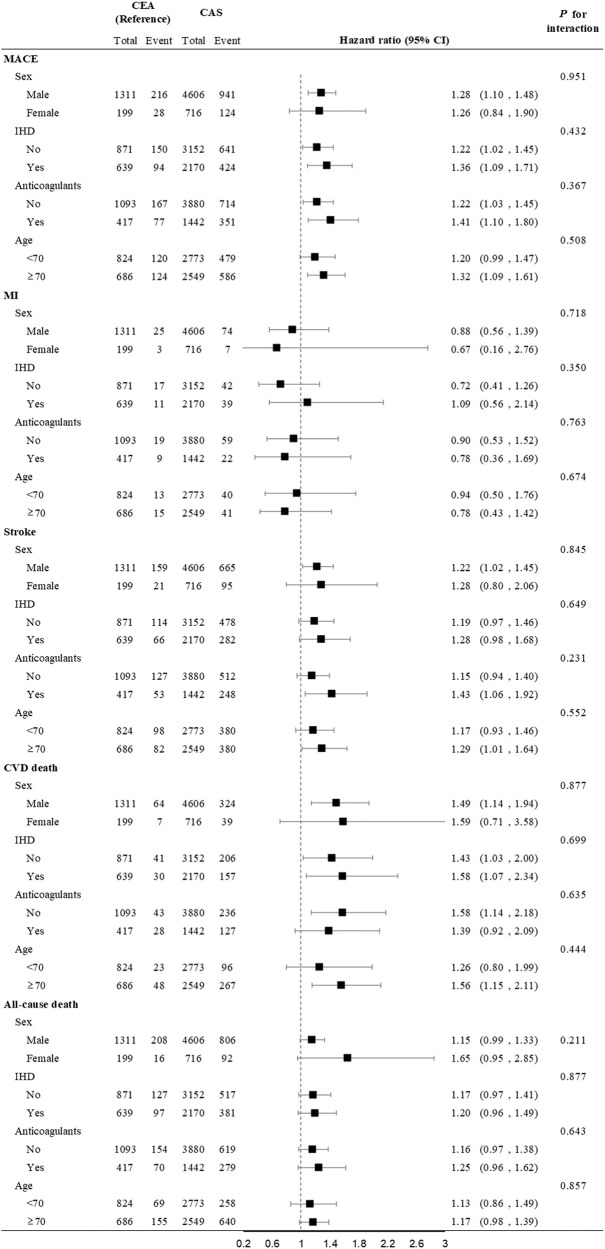
Forest plot of subgroup analysis with outcomes including MACE and all-cause death. MACE: major adverse cardiovascular event

When subgroup analysis was performed, ischemic heart disease and pre-procedure anticoagulation were found not to affect the incidence of MACE (P for interaction = 0.432 and 0.367, respectively). Old age (>70 years) did not affect the occurrence of MACE or stroke (P for interaction = 0.508 and 0.552, respectively), but CAS was associated with worse outcomes in this age category in terms of MACE, stroke, and CVD death (**Fig. 4**).

## Discussion

The findings of our study have revealed that the incidence of stroke is higher during the periprocedural period (within 1 month) in symptomatic patients who underwent CEA, but that the rates of mortality and CVD death were lower in both the early and long-term follow-up periods compared to symptomatic patients who underwent CAS. The results were similar in all patients, including both symptomatic and asymptomatic cases, who underwent a carotid revascularization procedure. However, the incidence of MACE and mortality rates did not differ between the CEA and CAS groups among asymptomatic patients.

As mentioned above, a number of RCTs comparing CEA and CAS in the treatment of carotid artery stenosis have been conducted, with some conflicting results.^5)^ A prior meta-analysis of 1-month outcomes in 10 RCTs comparing CAS and CEA found that CAS was associated with higher rates of any stroke.^10)^ In contrast, our study found that the CEA group in symptomatic patients had a higher incidence of stroke within 1 month. Similar results were also reported in another large-scale study of 430000 patients from the Nationwide Readmissions Database in the United States. In that study, CEA patients had a higher rate of periprocedural stroke than CAS cases but a lower rate of overall inpatient mortality.^11)^ These discrepancies may be due to various confounding factors. Compared with the results reported by the CREST trial (Carotid Revascularization Endarterectomy Versus Stenting Trial), a large RCT conducted in 2010, the periprocedural symptomatic stroke risk with CEA in our study was considerably higher (3.2% in the CREST population versus 6.8% in our cohort).^3)^ The periprocedural stroke incidence of CEA may be largely influenced by intra-procedural complications, including technical errors.^12)^ The poor 1-month stroke incidence of CEA observed in our cohort might have been due to the objectively higher procedural stroke risk in the CEA group. These early poor outcomes of CEA in our series may be attributed to a lack of trained vascular surgeons and the limited number of high-volume vascular centers in Korea. As of 2022, according to the Korean Surgical Society, there were only 138 authorized vascular surgeons in Korea. Several previous studies have suggested that higher surgical volume is associated with lower complication rates in CEA and that the surgeon’s specialty may also influence outcomes.^13–15)^ The improved outcomes in terms of 30-day mortality and stroke rates in our CAS patients may have contributed to the superior early outcomes in this group compared to the CEA group. Other studies have reported that the 30-day mortality and stroke rates following CAS have improved over the past 10 to 15 years.^11,16,17)^ Similarly, in our study, the periprocedural stroke rates in symptomatic patients who underwent CAS were lower than those reported in the CREST study (5.5% vs. 4.2%).^3)^ In conclusion, it is believed that the poor outcomes of CEA, driven by the shortage of vascular surgeons in Korea, along with improvements in CAS outcomes, contributed to the higher early stroke risk observed in our study. However, in terms of 1-year outcomes, the stroke risk was not significantly different between the 2 study groups, consistent with findings from other studies. In a pooled analysis of the 4 largest RCTs, the long-term incidence of stroke, beyond the periprocedural period, was found to be similar between the 2 revascularization procedures.^18)^

Unlike stroke risk, the rates of all-cause death and CVD death were higher in our symptomatic CAS group during both the early and long-term follow-up periods. These findings are consistent with previous RCTs, which reported that CAS was associated with higher post-procedural rates of death and MI, and that periprocedural MI contributed to increased long-term mortality.^10)^ The higher mortality observed in our symptomatic CAS group may be attributed to the relatively higher pre-procedural comorbidity among these patients. Although no significant differences were observed in the baseline characteristics between the 2 groups in our study, the number of comorbidities included in our analysis was limited, making an accurate comparison of comorbidity and surgical risk between the 2 groups difficult. However, many clinicians generally prefer CAS over CEA in patients who are at high surgical risk.^19)^ Similarly, in a previous study, the proportion of patients with extreme or major mortality risk prior to the procedure was higher among CAS patients compared to CEA patients.^11)^

In asymptomatic patients, no significant difference was observed between the 2 carotid revascularization methods. These findings are consistent with those of a previous study using a German mandatory registry, which analyzed 104000 individuals and found no major difference in the risk of disabling stroke or death, or any stroke or death.^20)^ Therefore, the choice of revascularization method should be individualized for asymptomatic patients.^21)^

In further subgroup analysis stratified by age (≥70 years), we found that age was not a contributing factor to differences in outcomes between the CAS and CEA groups. However, in subjects older than 70 years, we observed that the incidence of MACE, stroke, and CVD death was higher in the CAS group. This result is consistent with previous findings from the Carotid Stenosis Trialists’ Collaboration, which reported that age >70 years is associated with higher perioperative stroke rates after CAS, but not after CEA. This may be due to an increased atherosclerotic burden, aortic arch calcification, challenges in vascular anatomy, and greater plaque vulnerability.^4,22,23)^

Another subgroup analysis showed no interaction effects of anticoagulant use between the 2 groups. Recently, several studies have reported the beneficial effects of anticoagulants in atherosclerotic disease.^24,25)^ Notably, the Cardiovascular Outcomes for People Using Anticoagulation Strategies (COMPASS) trial found that a daily combination of low-dose rivaroxaban and 100 mg aspirin significantly lowered the rate of stroke, MI, or cardiovascular death.^25)^ However, an analysis within COMPASS of 1919 patients with carotid disease revealed that a higher dose of rivaroxaban did not reduce major vascular events in these patients but increased the risk of major bleeding.^25)^ Similarly, our current subgroup analysis found that anticoagulant use did not impact outcomes between the CAS and CEA groups. Further trials on the use of anticoagulants for carotid stenosis are needed.

This study was a nationwide, population-based analysis using patients registered in the Korean NHIS database. Unlike previous large studies that analyzed only Western populations, our findings are likely applicable to other East Asian countries due to commonalities in several ethnic and demographic characteristics, such as lower BMI and the unique epidemiology of CVD, both of which differ from those in Western populations.^9,26)^ Therefore, our study provides valuable information on the clinical outcomes of carotid stenosis treated with 2 different revascularization methods in Asian populations.

The number of carotid revascularization procedures increased in Korea during our study period (2010–2018). The total number of procedures in our study series, including both CAS and CEA cases, rose from 349 in 2010 to 1564 in 2018. These findings may also be valuable for clinicians in developing countries where carotid revascularization cases are increasing. Lastly, with higher surgical volumes and growing surgeon experience, better clinical outcomes are expected, and further studies are needed to confirm these trends.

There were several limitations of our study. First, patients who underwent transcarotid artery revascularization with flow reversal (TCAR), a novel hybrid procedure, were not included in the current analysis because this treatment is not yet available in Korea. Although no RCTs have compared TCAR with other carotid revascularization methods, a systematic review of TCAR involving 18 observational studies reported low 30-day rates of stroke, MI, and death.^27)^ Second, several pieces of clinical information that could have impacted our findings were not included in the analysis due to data limitations in the Korean NHIS database. Specifically, we were unable to assess pre-procedural tandem lesions or the laterality of post-procedure stroke, which may have influenced outcomes such as stroke incidence and cardiovascular death.

## Conclusion

Our population-based analysis of patients in the Korean NHIS database found that in symptomatic carotid stenosis patients, the periprocedural stroke rate was higher after CEA, while the mortality rate was higher after CAS, both in the early postoperative stages and over the long term. In asymptomatic patients, however, the incidences of MACE and mortality were comparable between CEA and CAS. Further studies are needed as surgical volume increases and surgical/clinical experience with both CEA and CAS continues to grow.

## Declarations

### Consent for publication

This manuscript does not contain any individual person’s data in any form.

### Ethics approval and consent to participate

This study was conducted in accordance with the principles of the Declaration of Helsinki and was approved by the Ethics Committee of Korea University Anam Hospital (2020AN0279). Informed consent from patients was waived due to the retrospective design of the study.

### Funding

This study was supported by a grant from Asan Institute for Life Sciences and Corporate Relations of Asan Medical Center, Seoul, Korea.

### Data availability

The data that support the findings of this study are available from the corresponding author upon reasonable request.

### Author contributions

Data collection, data analysis, writing: SAL

Data collection, data analysis, writing: DHC

Data collection, data analysis: JMC

Study design, data collection, data analysis, writing: JGG

All authors have reviewed and approved the final version of this manuscript.

### Disclosure statement

The authors declare that they have no competing interests.
